# Are patents linked on Twitter? A case study of Google patents

**DOI:** 10.1007/s11192-022-04519-y

**Published:** 2022-10-10

**Authors:** Enrique Orduña-Malea, Cristina I. Font-Julián

**Affiliations:** 1 Department of Audiovisual Communication, Documentation and History of Art, Universitat Politècnica de València, Valencia, Spain; 2 Department of Communication, Universitat Pompeu Fabra, Barcelona, Spain

**Keywords:** Patents, Twitter, Social media metrics, Altmetrics, Link analysis, Social patentometrics

## Abstract

This study attempts to analyze patents as cited/mentioned documents to better understand the interest, dissemination and engagement of these documents in social environments, laying the foundations for social media studies of patents (social Patentometrics).Particularly, this study aims to determine how patents are disseminated on Twitter by analyzing three elements: *tweets* linking to patents, *users *linking to patents, and *patents *linked from Twitter*.* To do this, all the tweets containing at least one link to a full-text patent available on Google Patents were collected and analyzed, yielding a total of 126,815 tweets (and 129,001 links) to 86,417 patents. The results evidence an increase of the number of linking tweets over the years, presumably due to the creation of a standardized patent URL ID and the integration of Google Patents and Google Scholar, which took place in 2015. The engagement achieved by these tweets is limited (80.2% of tweets did not attract likes) but increasing notably since 2018. Two super-publisher twitter bot accounts (dailypatent and uspatentbot) are responsible of 53.3% of all the linking tweets, while most accounts are sporadic users linking to patent as part of a conversation. The patents most tweeted are, by far, from United States (87.5% of all links to Google Patents), mainly due to the effect of the two super-publishers. The impact of patents in terms of the number of tweets linking to them is unrelated to their year of publication, status or number of patent citations received, while controversial and media topics might be more determinant factors. However, further research is needed to better understand the topics discussed around patents on Twitter, the users involved, and the metrics attained. Given the increasing number of linking users and linked patents, this study finds Twitter as a relevant source to measure patent-level metrics, shedding light on the impact and interest of patents by the broad public.

## Introduction

The rise of the Altmetrics allowed measuring broader impact of scholarly publications (Adie, 2016; Holmberg, 2015; Sugimoto et al., 2017; Warren et al., 2017), expanding the cited publications to be analyzed (from journal articles to any scholarly output), the citing publications to be considered (from scholarly publications to non-scholarly publications), and the nature of the performance metrics available (from bibliographic citations to usage, dissemination, comments, discussion, rating or connectivity), leading to a new generation of research metrics (Orduña-Malea et al., 2016; Priem & Hemminger, 2010) led by social acts (Haustein et al., 2016a).

By embedding scholarly works or the online research objects representing them (e.g. the full-text article’s URL) on social applications and platforms (Haustein, Bowman and Costas, 2016a), meta-researchers were allowed to capture signals of the interest, dissemination and engagement of the research endeavor beyond the scholarly environment (Tahamtan & Bornmann, 2020). This way, the development of the Altmetrics field unraveled data prevalence differences between social media sources (Haustein et al., 2015; Thelwall, 2018), data aggregators (Zahedi, et al., 2015; Ortega, 2018a, 2018b, 2020), and data accumulation velocity differences (Fang & Costas, 2020), being those differences shaped by the characteristics of each discipline (Htoo & Na, 2017; Orduna-Malea & Delgado López-Cózar, 2019; Zahedi et al., 2014).

Patents have been scarcely studied within this social analytical framework. The role of patents in Altmetrics studies has been limited to being a source of citations (i.e. the citing publications) for the documents being monitored (i.e. the cited publications), in a similar way than the role played in Patentometrics classic studies, were references from patents to papers are analyzed (Hammarfelt, 2021). Earlier webometric studies on patents have also focused on URL mentions from patents to other online resources (Orduna‐Malea, et al., 2017; Font-Julián et al., 2022).

**Fig. 1:**
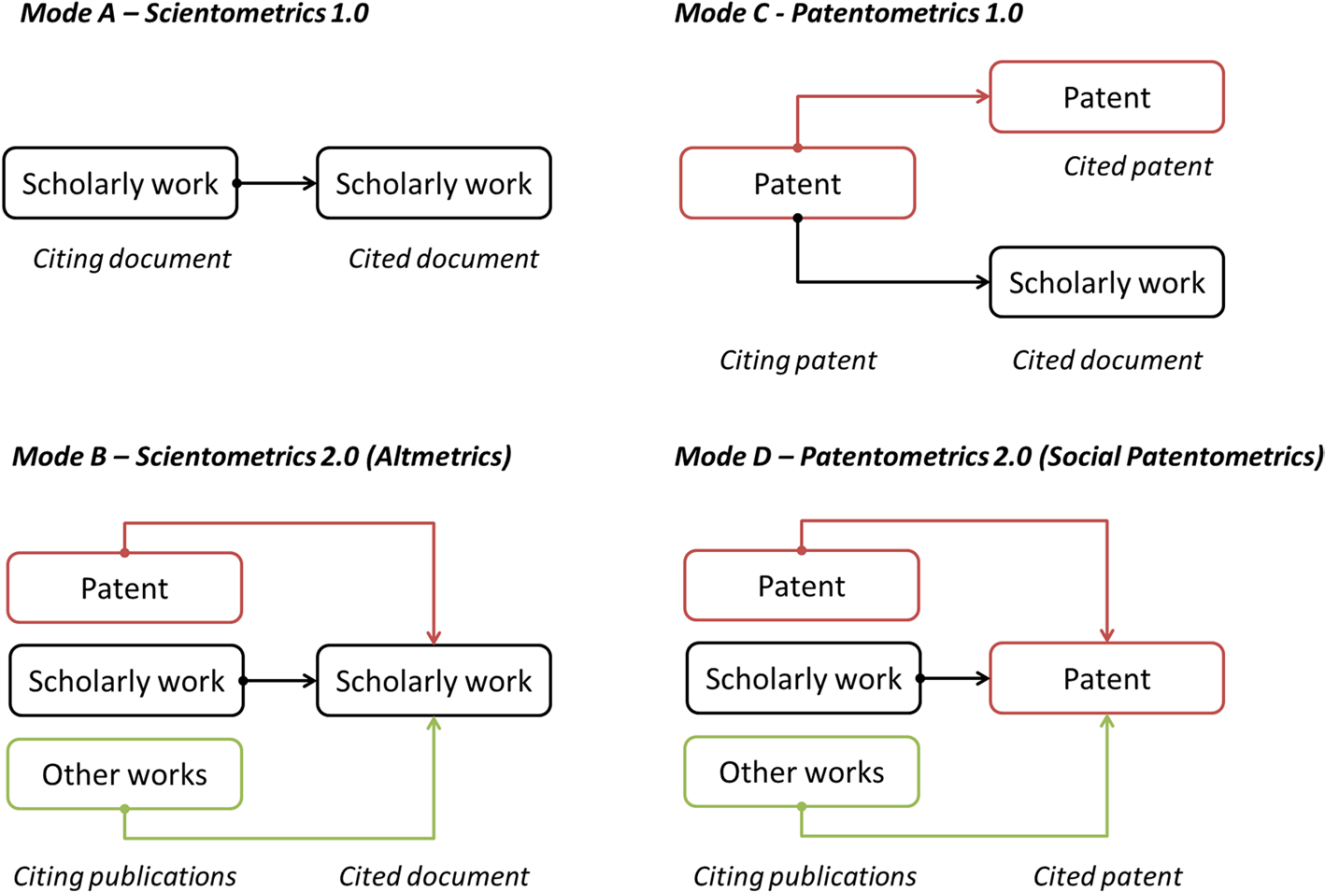
Basic models of Scientometrics and Patentometrics studies. Note: arrows connect citing documents to cited documents

Altmetrics reflects an evolution from Scientometrics 1.0 (Fig. 1; mode A) to Scientometrics 2.0 or social Scientometrics (Fig. 1; mode B). However, Patentometrics 1.0 (Fig. 1; mode C) did not evolve into a social Patentometrics model (Fig. 1; mode D). This contribution intends precisely to lay the foundations for social Patentometrics studies.

The lack of social media metrics studies of patents might be due to the following considerations:

### Purpose of patents

“A patent is a right granted to the owner of an invention that prevents others from making, using, importing, or selling the invention without the inventor’s permission” (Marley, 2014), limited to one specific jurisdiction during a limited period. Therefore, patents are legal documents, whose generation, consumption and impact are guided by dynamics different from those of the scholarly community.

### Access to patents

While patents are made available to the public at large as part of the disclosure obligation of inventors (Graham and Hedge, 2015), most web-based patent databases exhibit lack of advance search features (Marley, 2014). Likewise, other databases are offered by the patent office where the patents were submitted to request protection, limiting its coverage to that jurisdiction, and preventing thus the analysis of family patents (Martínez, 2011). In other cases, web-based patent databases offer only online descriptive metadata, with no online access to the full-text document.

### Interest on patents

As part of the disclosure obligation, inventors are required to accomplish a few conditions, which can vary slightly from one patent office to another. For example, patentees in the United States must satisfy three conditions (Ouellette, 2012): a *written description* (disclosing the technologic knowledge upon which the patent is based, and demonstrating that the patentee is in possession of the invention that is claimed), *enablement* (how to make and how to use the invention), and *best mode* (the patent must include “the best mode contemplated by the inventor or joint inventor of carrying out the invention”). This makes patents useful documents with technical solutions to problems, offering researchers both scientific and legal benefits for reading patents (Ouellette, 2017). However, the literature has also evidenced potential concerns related to the information offered. Patents might be perceived as hard to read and understand, vague, with extensive legal jargon included. In addition, there is a perception that reading patents might lead to increased liability for ‘willful’ patent infringement (Ouellette, 2012). These issues might show patents as less attractive documents for researchers (Lemley, 2008), making them underutilized scientific resources.

The subsequent emergence of open full-text patent databases accessible on the Web (each patent holds a URL) and covering documents from many patent offices around the world, enabled webometrics and Altmetrics studies to be carried out, allowing the social Patentometrics model (Fig. 1; mode D). These databases include patent search facilities (e.g. The Lens), patent search engines (e.g. Google Patents, Yandex Patents) and patent databases (e.g. Free Patents Online, Trea).

URLs linking to openly available full-text patents can be subsequently embedded on social media platforms (e.g. Twitter, LinkedIn), not only enhancing the dissemination of the patents but also allowing the capture of usage, interest and readership evidence, an aspect scarcely covered by the literature–basically through survey data (Ouellette, 2017), with no quantitative studies to date.

Among the currently available social media platforms, Twitter stands out due to its communicative nature and wide use. As of May, 2022, Twitter exhibits a high number of users (around 229 million monetizable daily active users) and tweets published (around 850 million tweets per day). Moreover, the existence of an Academic API and the availability of a wide variety of engagement metrics make Twitter a suitable platform to collect interest on patent publications.

**Fig. 2:**
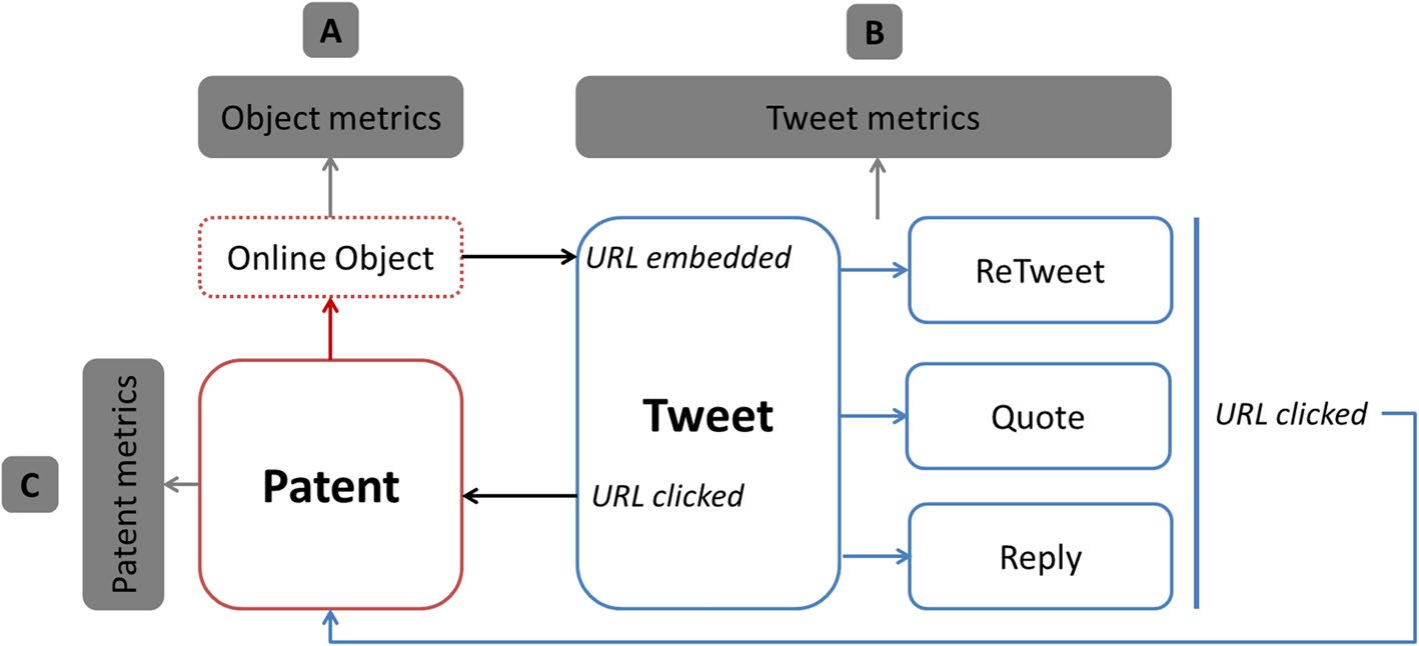
Role of Twitter in social patentometrics studies

Following in the model proposed by Fang et al. (2021), the Fig. 2 shows how Twitter can be employed to connect two landscapes: the innovation landscape and the Twitter landscape. Each online full-text patent holds a URL (research object) which can be embedded on one tweet. This tweet can be engaged by other users, who can like, retweet or reply to the original tweet. In addition, by clicking on the URL, users can be redirected to the patent. This model can collect three types of metrics: URL-based metrics (e.g. number of times a patent URL has been mentioned), tweet-based metrics (e.g. number of likes that a tweet mentioning patents has received), and patent-based metrics (e.g. number of visits that a patent has received from Twitter).

The main objective of this study is to disclose how patents are disseminated on Twitter. Specifically, this work focuses on analyzing three elements: *tweets* linking to patents (e.g. what volume of tweets is generated, what type of tweets are published, what impact do they generate); *users *linking to patents (e.g. what type of users link to patents, what is their activity on Twitter); and *patents *linked from Twitter (e.g. which patents are linked more frequently, which Patent Offices obtain a greater diffusion, which are the main subjects covered by the tweeted patents). This exploratory and descriptive research intends to lay the foundations for the future design of engagement metrics aimed at understanding the interest, dissemination, and engagement of patent documents in social environments.

To address that main objective, the following research questions are set:

- RQ1. What is the volume of tweets linking to patents?
- RQ2. What is the impact of tweets linking to patents?
- RQ3. What type of Twitter users link patents?
- RQ4. Which patents are most linked from Twitter?

To answer the research questions established above, the Google Patents database is used as case study. This decision is based on its wide global coverage of patents, its ease of use, and the generation of friendly URLs for each patent, which have made Google Patents one of the main patent discovery tools for researchers (Ouellete, 2015).

## Research background

### Google patents: a global online full-text patent discovery tool

Google Patents is a search engine and discovering tool launched in December 14, 2006, that indexes full-text granted patents and patents applications. As of May 2022, Google Patents covers over 140 million patent publications from 105 patent offices around the globe. Full-text documents are indexed from 22 patent offices.

Google Patents includes advanced search engines, global litigation information and a Prior Art Finder Tool, which includes a copy of the “technical documents and books indexed in Google Scholar and Google Books, as well as documents included in the Prior Art Archive”.^1^ For each patent, full-text, figures, the original PDF version, metadata, and citations are included. Patents with only non-English text have been machine-translated to English.^2^

While Google Patents is currently used as data source for Patentometrics studies and literature reviews (e.g., Narayanankutty, 2019, 2022), the literature aimed at describing and characterizing its features from an informational perspective has been limited. Noruzi and Abdekhoda (2014) and Marley (2014) described its search functionalities, and Moskovkin et al. (2012) showed Google Patents as a patent-metric tool useful to analyze the patent activity of world-wide leading innovation companies. Other works have explored Google Patents as a tool to find patent citations to scholarly works (Kousha & Thelwall, 2015) or URL citations to university websites (Orduna‐Malea, Thelwall and Kousha, 2017).

### Twitter: a social platform disseminating contents

Twitter is a real-time microblogging and social networking platform, launched in July 2006. Users can post brief plain text messages referred to as tweets, which can be liked or retweeted (with or without a quote) by other users. The platform collects metrics both at the user-level (e.g. number of followers, followings, tweets posted) and at the tweet-level (e.g. number of likes or retweets received) along with a wide variety of descriptive contextual metrics (e.g. tweet language, user location, etc.), making Twitter a potential tool to obtain scholarly metrics (Haustein, 2019).

Since the origin of Altmetrics, Twitter mentions have become one of the most important Altmetric events for scientific publications (Sugimoto et al., 2017), being the number of likes an important factor for measuring the social media activity of users around science (Díaz-Faes et al., 2019), although limitations such as the stability of twitter data (Fang et al., 2020a, 2020b) should be taken into account. Interactions between users and tweets around scholarly publications have been also analyzed (Friedrich et al., 2015; Haustein et al., 2015; Didegah et al., 2018; Hassan et al., 2021; ﻿Costas et al., 2021; Fang et al., 2021), including automatically generated content (Haustein et al., 2016b).

Despite the extensive literature focused on the scholarly use of Twitter, however, the dissemination and interest of patents in this social networking platform has not been studied to date.

## Method

### Tweets data collection

A python script was employed via the Academic Twitter API v2^3^ (full-archive search endpoint) to collect all tweets containing a URL to a full-text patent available on Google Patents, using the following ULR seed: ‘patents.google.com/patent/*’. The data collection expanded from 26 March 2006 (the birth of Twitter) to 31 December 2021. Data extraction was carried out by 23 February 2022. Original, reply and quoted tweets were considered, while retweets were excluded as these tweets are re-publications that can distort the results obtained.

For each tweet, the following parameters were captured: author id, username, tweet id, conversation id, public metrics (number of retweets, replies, likes, and quotes received), Twitter type (original, reply, quoted), and the tweet text. Results were obtained in JSON format, which were subsequently transformed into CSV files via OpenRefine to be further analyzed. This process yielded 126,815 tweets from 26,106 users.

### Accounts data collection

A second python script was built (Users by ID endpoint) to collect users’ descriptive data. For each Twitter user, the following data were captured: username, created at, lists, tweets, followers, and followings. Data was captured by 17 May 2022.

To characterize users’ behavior, the Botometer API^4^ was employed. Botometer is an application that scores Twitter users from 0 to 5 considering six variables,^5^ and using language-independence features (Sayyadiharikandeh, 2020). Scores near 5 reflect a bot-like behavior, while scores near 0 reflect human-like behavior. To do this, the most productive Twitter users (those who published at least 10 tweets linking to patents) were analyzed. While this threshold might be perceived as a subjective decision, these users (536) are responsible of the 70.1% of all tweets collected, constituting a representative sample. Data was collected by 24 May 2022, using the display universal score.

### Patents data collection

All URLs embedded in each of the 126,815 linking tweets were extracted, comprising 146,641 links, both to patents and other online resources. As Google Patents uses URL aliases,^6^ a data cleansing process was needed to identify the patents tweeted, by extracting the patent ID embedded in each Google Patent URL, which includes the country/Area code (patent office), number constitution, and kind code. All the type codes related to the same patent number constitution were combined to facilitate the analysis. This process yielded 86,417 patents.

The Patent API v.1.2.7 offered by The Lens^7^ was used to collect descriptive data related to each patent. The following parameters were considered: publication date, patent type, patent language, patent status, and patent citations. Data were collected by 27 May 2022.

## Results

### RQ1. What is the volume of tweets linking to patents?

**Fig. 3:**
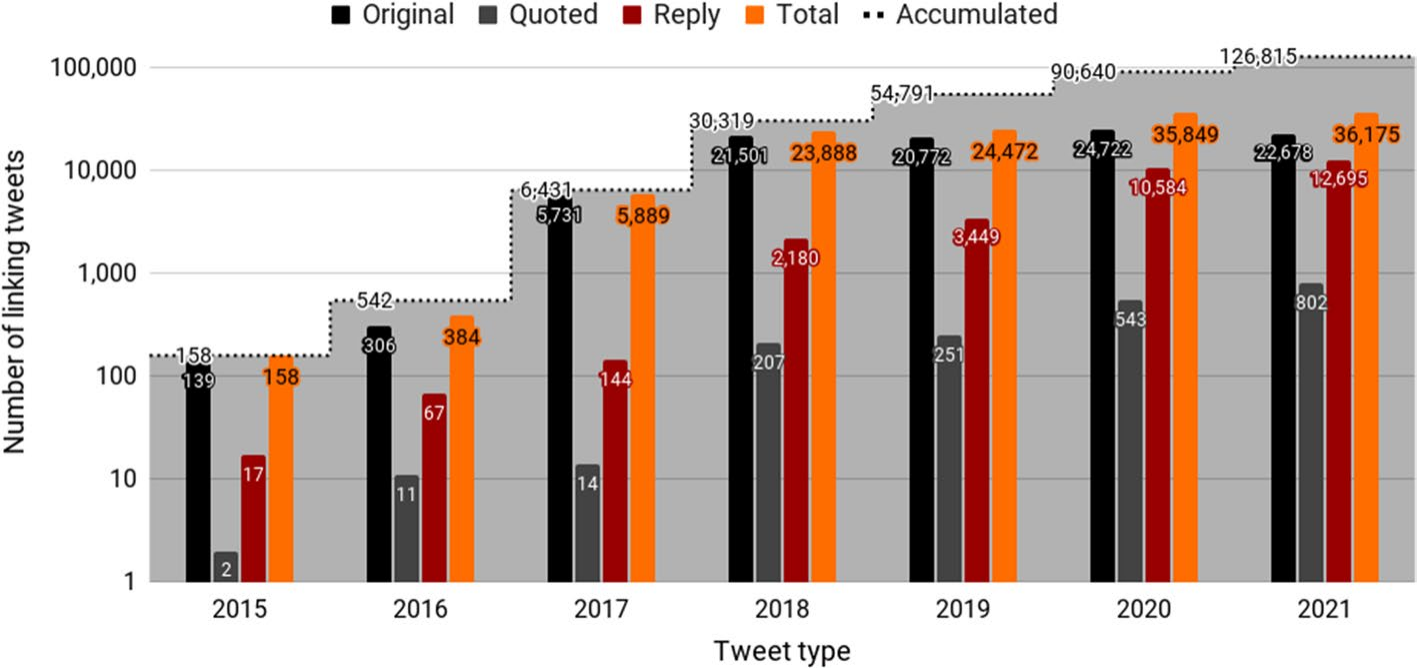
Number of linking tweets by tweet type over the years

The first tweet containing a link to a Google Patents’ full-text patent appeared in 2015, date that coincides with a significant update of Google Patents, including its integration with Google Scholar.^8^ Since then, 126,815 tweets have been published (Fig. 3), mainly original tweets (75.6%) and replies (23%). The year 2018 marks a milestone (23,888 tweets published), date that also coincides with another Google Patent’s update, in this case with the addition of global litigation information, via a partnership with Darts-IP,^9^ currently part of Clarivate.

### RQ2. What is the impact of tweets linking to patents?

The engagement received by tweets differs according to the type of tweet. Quotes and replies receive in average (arithmetic mean) more likes and retweets than original tweets (Table 1), which might imply that tweets around conversation generate more interest on users. Due to the skewed data, geometric means are also offered. In this case, we observe that original tweets improve their engagement notably. However, as the geometric mean operates only with values greater than 0 (i.e. eliminating all tweets with no engagement), the results can be misleading and should be taken cautiously and jointly with the arithmetic means obtained.

**Table 1:** Average engagement metrics according to the tweet type (original, reply, quote)

Engagement metric	Tweet type					
	Quote		Reply		Original	
	Arithmetic Mean	Geometric Mean	Arithmetic Mean	Geometric Mean	Arithmetic Mean	Geometric Mean
Retweet counts	1.7	2.03	0.6	1.80	0.5	2.02
Reply counts	0.5	1.26	0.5	1.17	0.1	1.49
Like counts	4.9	2.78	2.1	2.01	1.3	2.26
Quotes counts	0.3	1.16	0.1	1.21	0.1	1.40

A Mean: Arithmetic mean; G mean: Geometric mean (only values greater than 0)

The prevalence of the engagement metrics is limited (Table 2). Only 19.8% of all tweets linking to full-text patents have attracted at least one like. Likewise, 12.8% of tweets have received at least one reply, 9.1% of tweets have attracted at least one retweet, and 3.4% of tweets have received at least one quote. These percentages, however, have annually increased since 2017.

**Table 2:** Annual engagement metrics: likes, retweets, replies and quotes

	Year	Prevalence (%)	i1	i10	i100	Max	Interactions	Rate
Retweets RT	2015	22.2	35	2	0	36	120	0.76
	2016	28.6	110	14	3	294	1,152	3.00
	2017	3.0	179	22	5	193	1560	0.26
	2018	6.6	1584	154	24	821	11,416	0.48
	2019	7.5	1834	135	17	919	11,168	0.46
	2020	8.9	3196	223	14	3033	15,555	0.43
	2021	12.6	4544	378	42	870	26,970	0.75
	All years	9.1	11,482	928	105	3033	67,941	0.54
Replies RP	2015	20.9	33	0	0	5	45	0.28
	2016	17.2	66	0	0	9	104	0.27
	2017	2.4	144	4	0	31	249	0.04
	2018	6.4	1525	18	0	77	2626	0.11
	2019	9.8	2392	36	1	130	4196	0.17
	2020	15.2	5445	47	0	75	8,356	0.23
	2021	18.3	6603	70	1	234	10,788	0.30
	All years	12.8	16,214	177	2	234	26,364	0.21
Likes LK	2015	36.7	58	3	0	35	165	1.04
	2016	38.3	147	17	2	253	1290	3.36
	2017	5.2	304	37	5	1229	3346	0.57
	2018	12.6	3017	297	40	1322	24,244	1.01
	2019	17.2	4218	399	42	1986	33,026	1.35
	2020	20.7	7430	663	57	7803	52,994	1.48
	2021	27.6	9972	1035	119	1862	79,996	2.21
	All years	19.8	25,166	2471	265	7803	195,061	1.54
Quotes QT	2015	0.0	0	0	0	0	0	0.00
	2016	5.7	22	0	0	7	36	0.09
	2017	1.1	66	14	0	12	101	0.02
	2018	2.4	573	16	1	119	1432	0.06
	2019	2.5	600	14	0	68	1241	0.05
	2020	3.7	1328	30	1	196	2321	0.06
	2021	4.7	1691	15	0	80	3229	0.09
	All years	3.4	4296	92	2	196	8360	0.07

Prevalence (%): percentage of linking tweets with at least one retweet/reply/like/quote each

i1: number of linking tweets with at least one retweet/reply/like/quote each

i10: number of linking tweets with at least ten retweets/replies/likes/quotes each

i100: number of linking tweets with at least 100 retweets/replies/likes/quotes each

Max: highest number of retweets/replies/likes/quotes achieved by a linking tweet

Interactions: sum of the number of retweets/replies/likes/quotes

Rate: Interactions/total number of linking tweets

All the engagement metrics measured show skewed distributions. The median values for all metrics and all years are zero. This way, few tweets achieve outstanding values (e.g. only 265 tweets attract more than 100 likes; only 105 tweets attract more than 100 retweets; and only 2 tweets attract more than 100 replies or quotes), while most tweets attract low interest: 12,819 tweets (50.9% of all tweets receiving likes) attract only one like.

The correlation (Spearman) between the engagement metrics is statistically significant, but moderate. Only the number of likes and retweets achieve a moderate positive correlation (R_s_ = 0.54; p-value: < 0.0001; α > 0.05) (Table 3a). When data is restricted to tweets with at least 10 likes received (Table 3b), results improve slightly, especially the correlation of quotes with the remaining metrics.

**Table 3:** Correlation (Spearman) between engagement metrics

(a) Full dataset (N = 126,815 tweets). All tweets				
Variables	Retweet count	Reply count	Like count	Quote count
Retweet count	1	0.30*	0.54*	0.34*
Reply count	0.30*	1	0.41*	0.24*
Like count	0.54*	0.41*	1	0.31*
Quote count	0.34*	0.24*	0.31*	1

(b) Selected dataset (N = 2,471 tweets). Tweets with a likes count > = 10				
Variables	Retweet count	Reply count	Like count	Quote count
Retweet count	1	0.29*	0.63*	0.54*
Reply count	0.29*	1	0.45*	0.39*
Like count	0.63*	0.45*	1	0.45*
Quote count	0.54*	0.39*	0.45*	1

*Significance level α > 0.05

**Fig. 4:**
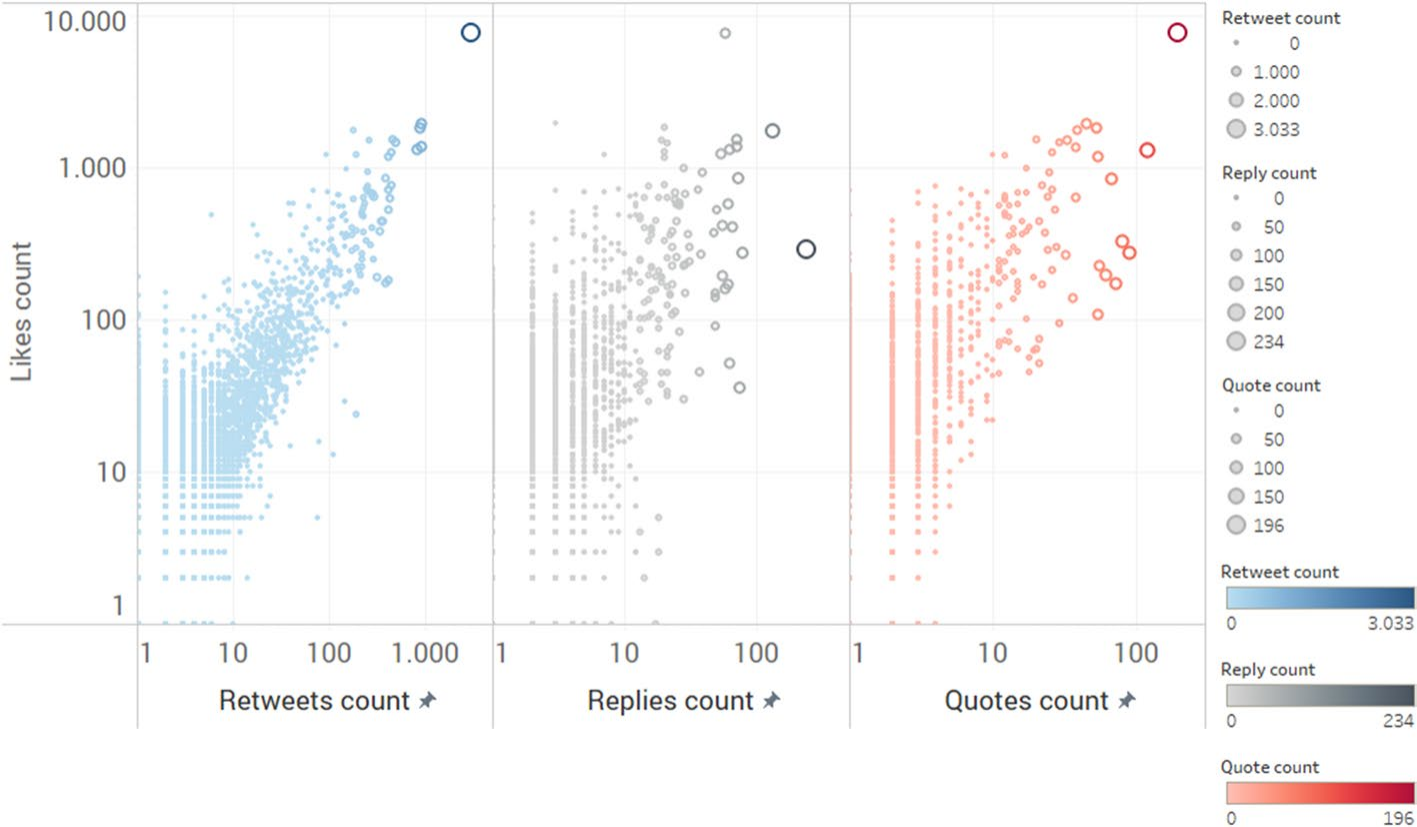
Scatter plot for engagement metrics: links count, retweets count, replies count, quotes count

A plausible reason for the low correlation values obtained is the low prevalence of the engagement metrics, previously observed. The high number of tweets with 0 or 1 likes/retweets/replies/quotes distorts the correlations achieved. However, when the number of likes received achieves a threshold (around 10), the correlation between the numbers of likes and retweets increases (Fig. 4). This threshold effect is hardly noticeable for the number of quotes and replies, which seem to reflect a different engagement dimension.

### RQ3. What type of twitter users link patents?

**Fig. 5:**
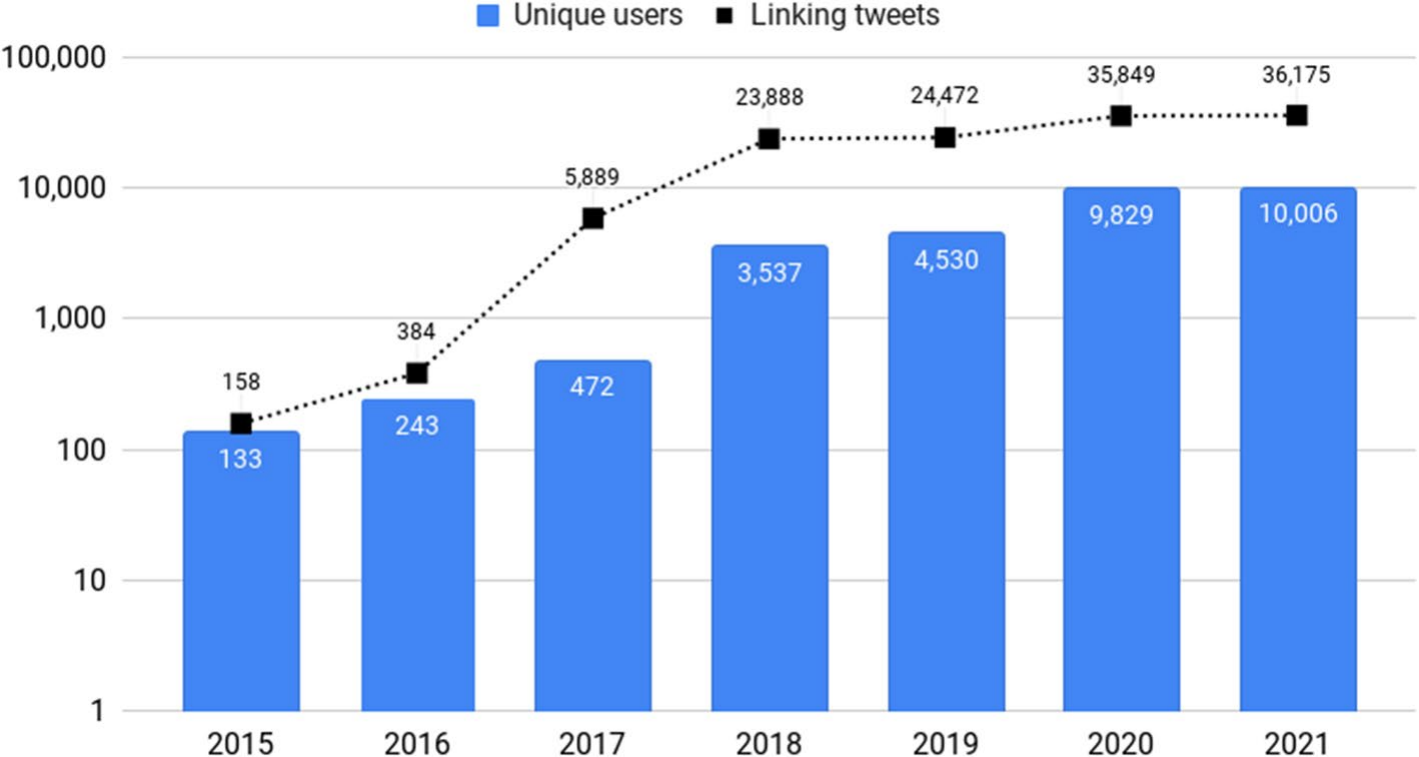
Number of Twitter users over the years

The 126,815 linking tweets have been published by 26,106 unique users. The number of unique users per year has increased notable since 2020. In 2021, a total of 10,006 unique users published 36,175 tweets linking to a full-text patent available on Google Patents (Fig. 5).

The distribution of linking tweets published per user is also quite skewed: 2 authors have published at least 100 tweets (high performers), while 19,656 users (75.3% of all users) have published only one linking tweet (sporadic users); 15.6% of all tweets come from sporadic users.

Two Twitter accounts (*DailyPatent* and *uspatentbot*) jointly publish 53.3% of all tweets (33,864 and 33,775 tweets, respectively), constituting the most influential users. These accounts are highly productive, do not follow other users, and most of their tweets published link to Google Patents (68.6% and 92.9%, respectively).

Considering the most productive users (Table 4), no specific characteristics regarding their behavior can be distinguished. We can find productive users with high number of followers and followings (e.g. *tatzanx*), productive users with high number of tweets published but few followers (e.g. *DinahParums*), productive users with low number of both followers and followings (e.g. *covidventilator*), or unproductive users with high number of followings (e.g. *PPAtrading*). *Patents_bot* and *patentsexpiring* are outlier accounts: all their published tweets link patents. In other cases, the linking patents amount for a low percentage of the total tweets published (e.g. *COILPOD*).

**Table 4:** Users publishing most linking tweets to Google Patents

User Name	Created at	Linking tweets	Total %	Individual %	Tweet count	Listed count	Followers count	Following count
DailyPatent	2016	33,864	26.7	68.61	49,357	9	72	1
Uspatentbot	2017	33,775	26.6	92.89	36,362	15	1678	0
Patents_bot	2017	1423	1.1	100	1423	14	118	0
Tatzanx	2009	1344	1.1	0.26	512,578	276	11,017	12,088
Covidventilator	2020	888	0.7	11.31	7849	0	35	3
Peaceful1979	2017	701	0.6	0.61	115,403	3	34	88
DinahParums	2013	580	0.5	0.25	234,414	21	0	734
ParolaAnalytics	2016	566	0.4	9.40	6023	18	903	1,117
SunstonePatents	2015	409	0.3	13.13	3115	19	388	149
micrornapro	2009	388	0.3	0.78	49,664	26	1709	4383
HumanInternet1	2019	387	0.3	7.44	5205	9	654	268
Ontrack9_oktogo	2021	338	0.3	6.83	4946	9	124	247
Jechepo	2010	274	0.2	70.62	388	1	31	218
Ogawa_tter	2011	272	0.2	0.43	63,922	162	3505	2064
estoppel88	2017	245	0.2	2.46	9952	17	1496	662
Patentsexpiring	2017	233	0.2	100	233	0	6	3
be4sure	2011	190	0.1	1.68	11,299	6	361	1,471
Housesitting15	2015	189	0.1	0.25	76,410	84	437	354
Intense_IP	2018	179	0.1	18.43	971	3	472	2320
Marie94167358	2020	177	0.1	2.47	7174	1	86	17
SyoK_PathLab	2020	173	0.1	3.06	5651	2	20	110
COILPOD	2013	157	0.1	0.37	42,777	0	1428	2323
Lennert_vd_Boom	2010	141	0.1	0.45	31,596	11	580	1068
Jaimecampos787	2016	130	0.1	1.95	6,679	20	451	701
ABTC_1	2018	128	0.1	0.38	33,958	9	1242	3247
NathanS27441765	2018	119	0.1	0.49	24,219	5	1437	4876
PPAtrading	2015	111	0.1	12.01	924	14	1000	4809
Notanwo	2020	110	0.1	2.43	4531	1	116	259
RobinsonMarabo	2021	109	0.1	3.51	3103	0	24	101
Bioactive	2008	106	0.1	0.42	25,483	22	541	1863
Abhilash_tard	2018	101	0.1	0.28	36,622	9	781	813
lfisk	2009	100	0.1	2.40	4,160	3	225	34

**Fig. 6:**
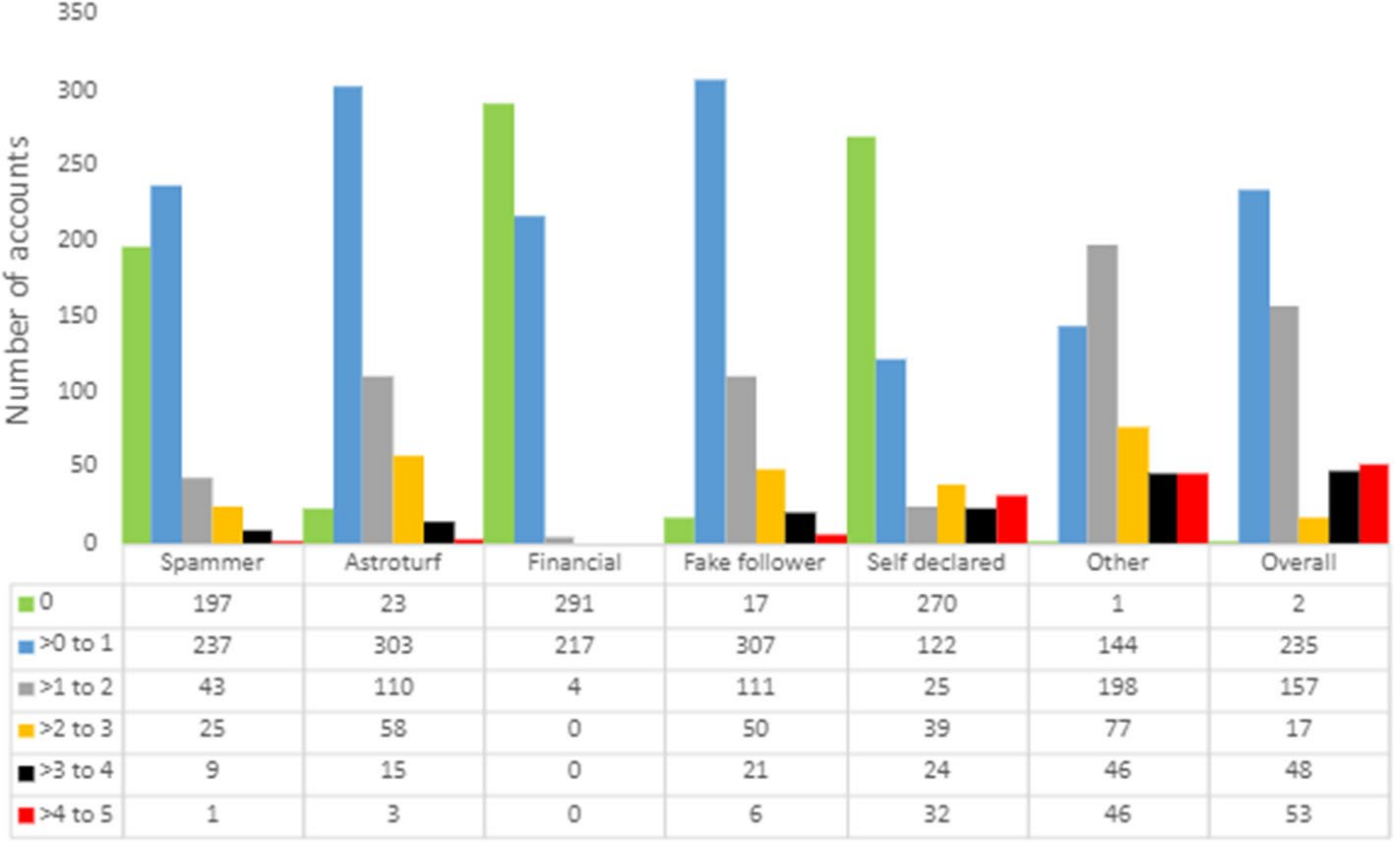
Types of users publishing most linking tweets to Google Patents (*N* = 512)**.** Note: each user can be categorized under more than one type

A deep analysis of 536 moderate and highly performers (those publishing at least 10 tweets linking to Google Patents) reveals an obsolescence of users (32 accounts have been suspended or eliminated at the time of the data analysis), a remarkable presence of bots (or human publishing like a bot), with 10% of those 504 active accounts exhibiting a Botometer display universal score higher than 4, of which 32 are self-declared bots (Fig. 6), and a high presence of individual (445) over organizational (59) accounts.

### RQ4. Which patents are most linked from twitter?

The United States Patent and Trademark Office (UPSTO) is the jurisdiction receiving most links from Twitter (112,949 links to 81,156 patents), followed at a great distance by the World Intellectual Property Organization (WIPO), the China National Intellectual Property Administration (CNIPA), European Patent Office (EPO) and the Japan Patent Office (JPO). The remaining jurisdictions are linked in a minority way. Otherwise, it is noteworthy the low number of links to patents from Denmark (8), Austria (5), Finland (3), Brazil (2) and Belgium (0) jurisdictions, whose patents are indexed full-text on Google Patents.

Table 5 includes the number of patents tweeted and the number of links to unique patents tweeted per patent office. Even considering that each Patent measured includes all the patent applications and granted patents related the same patent ID, the number of patents linked from Twitter constitutes a small percentage of the patents covered by Google Patents for each jurisdiction.

**Table 5:** Number of patents tweeted by patent office

Patent Office	Links to patents	Percentage of links (%)	Number of Patents	Percentage of Patents (%)	Grants	Applications
United States	112940	87.55	81,156	93.91	1,230,0193	6,876,815
WIPO	6196	4.80	1688	1.95	0	5,041,304
China	3930	3.05	621	0.72	23,163,083	14,561,559
EPO	2311	1.79	666	0.77	2,165,824	5,416,751
Japan	1709	1.32	1287	1.49	6,151,136	20,571,316
Canada	458	0.36	160	0.19	1,918,148	1,135,548
Germany	409	0.32	232	0.27	3,277,364	4,838,284
South Korea	399	0.31	220	0.25	2,852,423	3,920,608
Spain	172	0.13	56	0.06	950,507	1,052,013
United Kingdom	155	0.12	86	0.10	726,341	3,136,973
Russia	107	0.08	77	0.09	1,039,683	645,389
Australia	60	0.05	47	0.05	911,305	2,089,826
France	46	0.04	34	0.04	2,244,339	1,004,797
Taiwan	21	0.02	21	0.02	697,387	1,391,753
Switzerland	13	0.01	4	0.00	0	731,659
Netherlands	12	0.01	10	0.01	217,801	435,763

WIPO does not actually grant patents per se; the grant or refusal of a patent rests with the corresponding national or regional patent office

https://www.wipo.int/patents/en/faq_patents.html.

Human necessities (383 patents) and Physics (156 patents) are the subjects most covered by the patents most tweeted (those patents tweeted at least 5 times; *N* = 818). The presence of the remaining subjects is low (Table 6).

**Table 6:** Number of patents tweeted by Cooperative Patent Classification (CPC)

CPC Section	Scope	Number of patents	%
A	Human necessities	383	46.8
B	Performing operations; transporting	84	10.3
C	Chemistry; metallurgy	67	8.2
D	Textiles; paper	3	0.4
E	Fixed constructions	5	0.6
F	Mechanical engineering; lighting; heating; weapons; blasting engines or pumps	40	4.9
G	Physics	156	19.1
H	Electricity	77	9.4
Y	General tagging of new technological developments; general tagging of cross-sectional technologies spanning over several sections of the IPC; technical subjects covered by former USPC cross-reference art collections [XRACs] and digests	3	0.4

Only patents tweeted at least 5 times and indexed in The Lens (N = 818) are considered; one patent can include more than one category

**Fig. 7:**
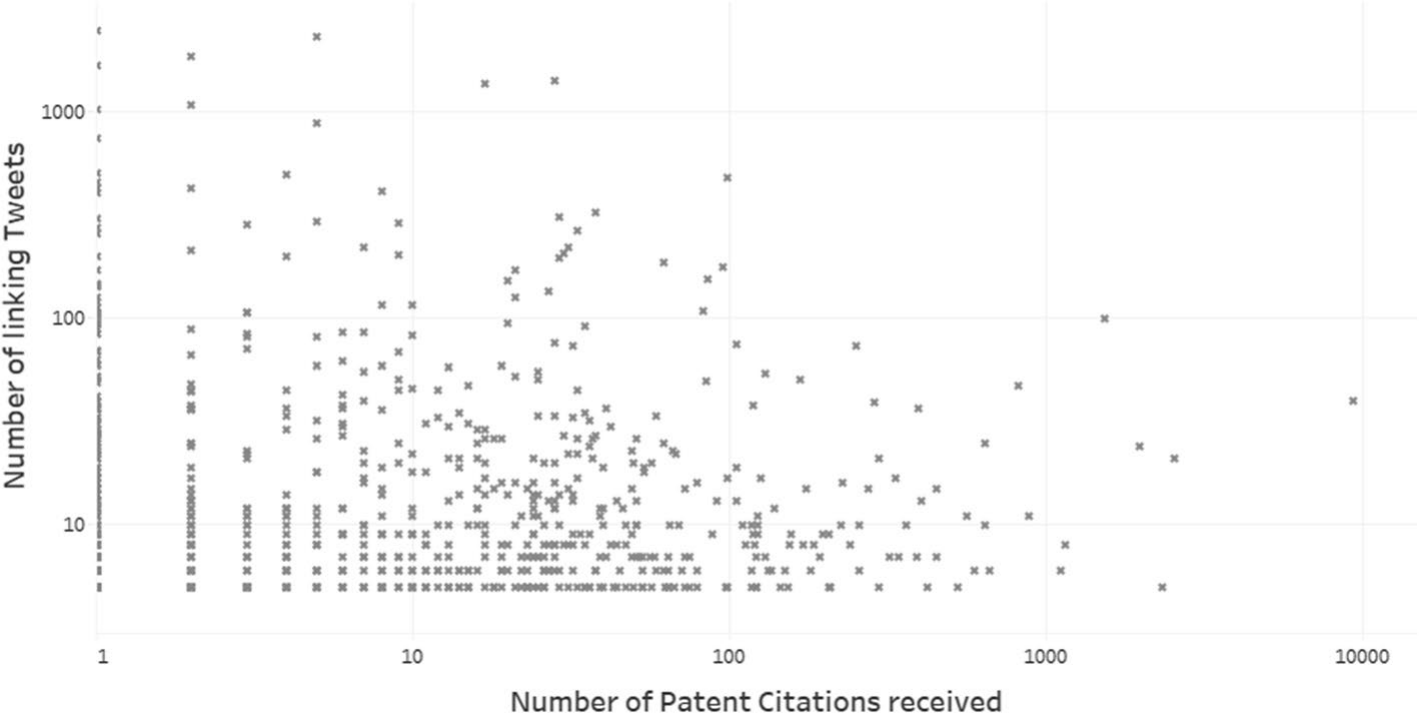
Scatter plot between the number of patent citations received and the number of tweets linking to the patent (*N* = 839)

A Chinese written patent application (CN112220919), related to covid-19 (with CPC code “A” [human necessities] assigned), published in 2021 and still pending, is the full-text patent most tweeted. This fact evidences that neither the time of publication, nor patent citations count or status are decisive variables in obtaining a higher dissemination on Twitter. Table 7 delve into this issue by displaying the top 20 tweeted patents, including the status, the publication date, the first CPC code, the number of patent citations (both from Lens and Google Patents), and the total number of citations (from Google Scholar). As we observe, highly tweeted patents include old/recent, active/expired or highly/lowly cited patents. Indeed, few highly cited patents have been highly tweeted (Fig. 7).

**Table 7:** Patents most tweeted

Patent	Tweets	Publication Year	Status	Patent citations (Lens)	Patent citations (GP)	All citations (GS)	CPC code
*CN112220919	2490	2021	Pending	0	0	0	A
US6506148	2329	2003	Expired	5	5	8	A
*US10130701	1874	2018	Active	2	8	2	A
WO2020060606	1677	2020	Pending	0	0	4	G
US5159703	1418	1992	Expired	28	21	38	H
*US7220852	1373	2007	Expired	17	17	12	C
*US20200279585	1079	2020	Active	2	1	0	G
US10144532	1030	2018	Active	1	2	3	B
WO2017115866	889	2017	Pending	5	3	NA	A
*EP3172319	744	2017	Active	0	7	2	C
US20060145019	505	2006	Abandoned	0	0	0	B
US10105389	496	2018	Active	4	5	1	A
US6630507	483	2003	Expired	99	81	94	A
*US11107588	455	2021	Active	0	0	0	G
*US7279327	429	2007	Expired	0	2	1	C
*EP1694829	425	2010	Active	2	31	NA	C
US6410059	410	2002	Expired	8	12	58	A
WO2019226774	404	2019	Pending	0	0	0	A
US3951134	327	1976	Expired	38	25	38	A
US5676977	309	1997	Expired	29	18	26	A

^*^Patents related to coronavirus

## Discussion

This study represents the first attempt of studying patents documents linked from Twitter. The linking tweets (volume, type, and impact), linking users (productivity, type, profile) and linked patents (jurisdiction, subject, and impact) have been determined and characterized. The main findings are discussed below.

### Volume of tweets linking to patents

The number of linking tweets is increasing since 2015, constituting a large-scale dataset for measuring purposes. The years 2020 and 2021 are remarkable with more than 30,000 linking tweets published in each of these years.

The increase of the overall volume of tweets over the years (in August 2013 were published around 500 million tweets per day; this figure has risen to around 867 million tweets in August 2022)^10^ and the launch of the bots *Dailypatent* and *uspatentbot* (created at 2016 and 2017, respectively) seem to be among the main Twitter-related causes of the take-off of tweets embedding links to Google Patents.

Beyond the Twitter activity, the integration of Google Scholar with Google Patents in 2015 might have also influenced the rise of linking tweets. Given the importance of Google Scholar as a discovery tool (Delgado López-Cózar et al., 2019), the use of this academic search engine could facilitate the discovery of patents, which users later spread through Twitter. These results are also aligned with the findings obtained by Ouellette (2017), who surveyed 832 US academic and industry researchers finding that 43% of these respondents found patents through Google Scholar, being this academic search engine the third preferred method to find patents after Google Patents (50%) and the USPTO website (45%). Therefore, we can infer that there was a latent interest in disseminating patents (the existence of over 127,000 tweets linking full-text patents available on Google Patents in 7 years might evidence that interest), and that the Google Scholar/Google Patents combination played a key role in the process of social dissemination of patents on Twitter.

A slight slowdown in the generation of linking tweets is detected in 2021. Therefore, it should be checked if after the covid-19 pandemic the number of linking tweets could be reduced, which seems likely given the remarkable number of coronavirus-related patents among the most tweeted patents (Table 7).

### Impact of tweets linking to patents

73.3% of all linking tweets (92,968) have obtained no engagement at all (zero likes, retweets, replies and quotes), while only the 1.6% (1,637) have obtained at least one interaction in each of the engagement metrics measured. This result is aligned with the user engagement found for 7,037,233 unique original scholarly tweets (Fang et al., 2022), where only the 2% of the tweets attained engagement in all the four types of user engagement.

The data prevalence for each of the engagement metrics achieves low percentages (see Table 2). Therefore, the results evidence a low impact, especially for original-type tweets. Reply-type tweets attain higher average engagements. For this reason, this type of tweets might be of great interest to locate and study conversations between Twitter users in which links to full-text patents are included as information resources.

The prevalence percentage of all the engagement metrics is increasing over the years. Hence, if the upward trend were to continue, the relevance of tweets to learn about the social dissemination of patents would increase. However, the skewed distribution found for all the engagement metrics (i.e. few tweets attract most of the engagement) could imply a high dependence on the overall impact of a few tweets linking few patents, which could have been mentioned for any reason. For example, the tweet 1313565051048128513, published in 2020, is the tweet most liked (7803 likes), retweeted (3033 retweets) and quoted (196 quotes) in the dataset, appearing in 127 linking tweets. This tweet embeds a URL to the patent US4656917A, a historic patent whose inventor is Van Halen, a famous guitarist who passed away that year. Likewise, the patent most tweeted in the dataset (CN112220919; embedded on 2490 tweets) describes the invention of a new coronavirus vaccine that contains graphene oxide, having generated extensive discussion and controversial both on and off Twitter, inside and outside the academic community.

The moderate correlations between the engagement metrics are partly caused by the effects of skewed distributions, that is, the high percentage of zero results, which should be avoided in overall descriptive analyses. Establishing a threshold (tweets with at least 10 likes received), the data shows a moderate significant positive correlation between the number of likes and retweets received (both reflecting a passive engagement; new content is not created). While the number of quotes received can be analyzed as an active engagement (i.e. new content is created), this metric is closer to the number of retweets received, which makes sense as the quote is a type of retweet (i.e. the users decide to quote when they are retweeting). Finally, the number of replies received (active engagement; new content is created) is only moderately correlated to the number of likes, reflecting a different engagement dimension in the corpus of tweets analyzed.

Given the higher engagement of the quote-type tweets, and the different behavior of the number of replies, the results indicate that metrics related to conversations could be especially relevant when it comes to better understanding the spread of patents on Twitter and developing impact metrics, while likes and retweets counts, despite being more numerous, could be less relevant when estimating impact.

If we take into account the total number of interactions received, likes (195,061) and retweets (67,941) are the most numerous metrics, while the number of replies (26,364) and quotes (8,360) are less used. These results are aligned with the engagement behavior found for scholarly tweets by Fang et al. (2022).

As regards the interaction rate, patents show low values: 1.54 likes per tweet and 0.54 retweets per tweet. These results are lower than those found by Fang et al. (2022) for research publications (2.95 likes per tweet and 1.91 retweets per tweet, respectively). While these results might evidence that patents are less engaged than scientific publications on Twitter, further research is deemed necessary to confirm this issue, as Fang et al. (2022) only managed publications indexed in Web of Science, and our contribution only analyzed patent IDs belonging to Google Patents. In any case, direct comparisons between patents and research publications should be discussed cautiously, as their communities of attention can overlap to some extent, but they are not necessarily the same.

### Users linking to patents

Most users are sporadic (75.3% of all users published only one linking tweet), being responsible of a low percentage of all the published tweets (15.6%). Therefore, it is plausible to infer that the approach of these users to the dissemination of patents is limited and probably due to the specific topic of one patent or their relation to that patent (i.e. the user is the inventor), an aspect that should be studied in greater detail.

If we focus on the few highly performer users, most of them are individual accounts but the most productive are institutional accounts publishing as bots; around 50% of all linking tweets come from two bots. Although bots are not necessarily negative (they post many tweets, generate dissemination, and encourage conversation), this data confirms that the dissemination of patents on Twitter is fundamentally through automated accounts and not individual users who share patents to disseminate its contents, foster its dissemination or discuss about specific topics in which the patent becomes a relevant information resource in the conversation.

Other findings have revealed a remarkable instability of productive users (deleted or suspended accounts), an issue already detected for tweeted publications (Fang et al., 2020a, 2020b), and an increase of unique users in 2020 (from 4,530 users in 2019 to 9,829 users in 2020; see Fig. 5), which might be an effect of specific conversations around polemic topics (e.g., coronavirus vaccination). Future studies should also check whether the number of unique users linking to patents might decline after the pandemic.

Otherwise, the activity of the most productive users does not follow specific patterns. The number of followers, followings, total tweets published is quite different among these users. In other words, the users who tweet many patents do not have a defined social profile.

### Volume of patents linked from Google patents

It was decided to combine all the different patent applications and granted patents registered under the same kind code as a unique patent ID. Even though there may be slight differences (e.g., publication date, claims) between these documents, they all refer to the same invention within the same jurisdiction. Therefore, the number of patents reported (86,417) should be understood at the level of the invention instead of the document.

While the combination of patent applications under the same kind code does not make it possible to calculate exact percentage values (the number of unique patent IDs is unknown), the percentage of patents tweeted is extremely low (results in Table 5 evidence this issue), given that Google Patents coverage is currently around 140 million documents (grants and applications). Taking this into account, the percentage of patents tweeted is around 0.06%, this value being an underrepresentation of the real value, which is estimated to be slightly higher.

Fang et al., (2020a, 2020b) reported altmetric data for “nearly 12.3 million Web of Science publications published between 2012 and 2018”, of which 34.01% had been mentioned on Twitter. Even though these percentages vary according to the Altmetrics aggregator, the temporal coverage of publications collected, the selected bibliographic database, and the promotion actions carried out by publishers, arguably, patents are less tweeted than publications.

The scarce number of patents tweeted along with their low impact might compromise the wide usage of social Patentometrics. A plausible reason is that researchers and publishers are interested in promoting their publications (Sugimoto et al., 2017). However, inventors do not follow this same rationale to promote patents, which could explain the large number of tweets coming from information services (many of them bots).

### Patents most linked from twitter

The jurisdictions whose patents are full text indexed by Google Patents are those receiving most links from Twitter, especially USPTO patents. However, these results are biased due to the behavior of the accounts *DailyPatent* and *uspatentbot*, which jointly publish 53.3% of all linking tweets (Table 4), and which only tweet US patents.

The patent most tweeted comes from the Chinese Patent Office, being related to the covid-19. This issue also evidences the coronavirus effect on the dissemination of patents on Twitter, as 8 out of the 20 patents most tweeted are related to covid-19. This result reflects the importance of specific events in generating patent outreach on Twitter. Other variables such as the number of patent citations received (a signal of the relevance of the patent) do not correlate with the number of tweets linking the patent.

Otherwise, discrepancies in patent citation counts between Lens and Google Patents have been noticed for specific highly tweeted patents. A larger scale analysis should be carried out to check the correlation between these sources. As regards Google Scholar citation counts, the results reveal inconsistencies. Google Scholar computes citation counts for patents considering both patent citations and non-patent citations. However, the results obtained for highly tweeted patents show inconsistent results, which should also be checked. However, the unavailability of an API for Google Patents makes this task difficult.

### Limitations

The results have shown a wide time lag between the appearance of Twitter and Google Patents (2006) and the appearance of the first tweet with a link to Google Patents (2015). In order to explain this late occurrence, the Internet Archive’s Wayback Machine^11^ was used to check the operating of Google Patents since 2006 to 2015, discovering that other URLs were used as patent URL IDs in the early years of Google Patents, depending on the Google market, such as ‘google.com/patents/about?id = *’ or ‘google.es/patents/about?id = *’.

To check the effects of these URLs on the results, all tweets containing the seed “google.com/patents/about?id = *” were collected. As we observe in Table 8, only 278 tweets were found from 2009 to 2014. Considering that ‘google.com’ is the most important Google’s domain name, we estimate the volume of linking tweets very low.

**Table 8:** Number of tweets linking the old patent URL ID (google.com/patents/about?id = *)

Year	Number of linking Tweets
2009	10
2010	58
2011	146
2012	47
2013	15
2014	2
2018	1
Total	279

All links using the old patent URL ID are currently broken and do not provide access to patent documents. In any case, the results reported in this work are limited to tweets from 2015 to 2021, as the URL seed analyzed was not active before 2015.

This fact reflects the limitation of using URL seeds (regular expressions) to collect linking tweets to patents. Moreover, future Google Patent website changes might create new URL IDs, making the data collection process complex. Still, this is the most effective method to collect the linking tweets because patents, unlike scientific publications, do not use standardized URL-based IDs, such as DOIs.

All the search queries performed have relied on the Academic Twitter API v2. Despite the service is quite efficient, recovery for all existing tweets for each query is not 100% guaranteed as “the Search API is focused on relevance and not completeness”, and some tweets (mainly spam, duplicate tweets or offensive tweets) may be missing from search results (Thelwall, 2015). Although this circumstance is explicitly pointed out by Twitter API v1^12^ and this work uses API v2, it is likely that this same problem would occur. In any case, the loss of these tweets is unlikely to be problematic.

Bots have been identified via the Botometer application. Therefore, the results are limited to the accuracy of this tool. The identification of bots is a complex task as human users can tweet “like a bot”. Therefore, there is a margin of error for those accounts that do not define themselves as bots.

Finally, patents have been analyzed through Google Patents. However, other patent databases offer free online access to patent documents. Consequently, one same patent can be linked from Twitter via different URLs. Hence, all results reported should be limited to patents indexed Google Patents in particular, not to patent applications in general.

### Future research

Even though this work has revealed general aspects about the dissemination and impact of patents on Twitter, the design of impact indicators at the patent level still requires a better understanding of the factors and variables involved in this communication process. Future lines of work in progress are indicated below:

#### Data inside the tweet

The analysis of the linking tweet text deems necessary to better understand the context in which the patent is being discussed or shared. Both quantitative (e.g. user mentions counts, hashtags counts, co-linked URLs counts) and qualitative (e.g. purpose of tweets, sentiment) should be carried out.

#### Data about the user

Each user linking to patents should be deeply described. For example, determining its role (inventor) or profession (industry, scholar, media, government, etc.) might provide new insights to better understand who and why tweet to patents. While pioneer studies aimed to characterize scholars on Twitter has been carried out (Costas et al., 2020; Mohammadi et al., 2018; Yu et al., 2019), there is a gap in the literature on industry researchers and users related to innovation on Twitter. In addition, given the predominant role of bots tweeting to patents, future studies should analyze the activity and impact of bots and humans separately.

#### Data about the engagement metrics

The engagement speed (e.g. the percentage of engagement received after 24 h since the publication of the linking tweet) and spread (e.g. the number and type of users engaging with the linking tweet, along with the languages used by these users and their origin) will allow a better understanding of the sensitivity and nature of the metrics, and therefore the design of more accurate impact indicators.

#### Data beyond Google patents

This work has analyzed one full text patent database. Future studies should consider other databases offering free full text access to patents (e.g. The Lens, Trea) to get a more comprehensive view of patents dissemination on Twitter, and other social media platforms (e.g. LinkedIn).

## Conclusions

Twitter has been proved to be a relevant venue for disseminating patents and inventions in a large non-academic setting. This study has unraveled the volume of tweets linking to full-text patents, the impact of these linking tweets, the main Twitter users linking to patents as well as the patents most linked. These findings have allowed laying the foundations for social Patentometrics, in which patents are placed as linked/mentioned online resources in social media.

Even though more studies are needed to understand the mechanisms that regulate the dissemination and consumption of information related to patents on Twitter, this study has made it possible to determine that the existence of discovery tools (Google Scholar) and full-text online databases (Google Patents) were necessary to enhance the dissemination of patents.

The results obtained also allow obtaining metrics related to the interest on patents. Despite the metrics analyzed in this study are indirect and based on a first generation of metrics (engagement with the tweet instead of the patent itself), the use of second-generation metrics (clicks on links) as well as web usage data on patent visits/downloads will provide more evidence of the use of patents by different types of audience, including academic and industrial researchers, practitioners, and the broad public.

## Footnotes

1 https://www.priorartarchive.org.

2 https://support.google.com/faqs/answer/7049585?hl=es&ref_topic=6390989.

3 https://developer.twitter.com/en/products/twitter-api/academic-research.

4 https://rapidapi.com/OSoMe/api/botometer-pro/details.

5 fake_follower: bots purchased to increase follower counts; self_declared: bots from botwiki.org; astroturf: manually labeled political bots and accounts involved in follow trains that systematically delete content; spammer: accounts labeled as spambots from several datasets; financial: bots that post using cashtags; other: miscellaneous other bots obtained from manual annotation, user feedback, etc.

6 The following URL were found: patents.google.com, google.co.uk/patents; google.co.ug/patents google.com.ar/patents, google.com.au/patents; google.com.br/patents; google.com.gi/patents; google.com.na/patents; google.com.pg/patents; google.com.tr/patents; google.de/patents; google.es/patents; google.ch/patents; google.ca/patents.

7 https://www.lens.org/lens/user/subscriptions#patents.

8 https://publicpolicy.googleblog.com/2015/07/good-patents-support-innovation-while.html

9 https://clarivate.com/darts-ip

10 https://www.internetlivestats.com/twitter-statistics/.

11 https://archive.org/web/.

12 https://developer.twitter.com/en/docs/twitter-api/v1/tweets/search/overview.
